# A Molecular Basis for Reciprocal Regulation between Pheromones and Hormones in Response to Dietary Cues in *C. elegans*

**DOI:** 10.3390/ijms21072366

**Published:** 2020-03-29

**Authors:** Saeram Park, Jun Young Park, Young-Ki Paik

**Affiliations:** Yonsei Proteome Research Center, Yonsei University, Seoul 03722, Korea; parksr@proteomix.org (S.P.); parkjy@proteomix.org (J.Y.P.)

**Keywords:** *C. elegans*, ascaroside, dafachronic acid, pheromone, hormone, development

## Abstract

Under stressful conditions, the early larvae of *C. elegans* enter dauer diapause, a non-aging period, driven by the seemingly opposite influence of ascaroside pheromones (ASCRs) and steroid hormone dafachronic acids (DAs). However, the molecular basis of how these small molecules engage in competitive crosstalk in coordination with insulin/IGF-1 signaling (IIS) remains elusive. Here we report a novel transcriptional regulatory pathway that seems to operate between the ASCR and DA biosynthesis under *ad libitum* (AL) feeding conditions or bacterial deprivation (BD). Although expression of the ASCR and DA biosynthetic genes reciprocally inhibit each other, ironically and interestingly, such dietary cue-mediated modulation requires the presence of the competitors. Under BD, induction of ASCR biosynthetic gene expression required DA, while ASCR suppresses the expression of the DA biosynthetic gene *daf-36*. The negative regulation of DA by ASCR was IIS-dependent, whereas *daf-36* regulation appeared to be independent of IIS. These observations suggest that the presence of ASCR determines the IIS-dependency of DA gene expression regardless of dietary conditions. Thus, our work defines a molecular basis for a novel reciprocal gene regulation of pheromones and hormones to cope with stressful conditions during development and aging.

## 1. Introduction

Animals have adapted to survive by overcoming environmental stresses in various ways. The nematode *Caenorhabditis elegans* secretes ascaroside pheromones (ASCRs); elevated pheromone levels are strong cues that induce dauer development under stressful conditions (e.g., starvation, overcrowding and high temperature) [1,2,3]. When worms perceive secreted ASCRs, the pheromone signals are integrated in sensory neurons (e.g., ASI neurons) and further transduced to trigger the appropriate responsive behaviors (e.g., avoidance, repulsion) or a developmental switch to dauer entry in early larvae (L1–L2) [3,4,5]. Since the first ASCR (daumone) was discovered [6], more than 160 ASCRs have now been identified [7,8,9,10,11]. These ASCRs are biosynthesized in part through a peroxisomal fatty acid β-oxidation pathway where several genes (e.g., *acox*, *maoc-1*, *dhs-28* and *daf-22*) are involved in the degradation of very long chain fatty acids into short chain fatty acids [8,10,12,13,14]. Due to its association with fatty acid metabolism, ASCR biosynthesis is usually activated by food abundance [15].

**Figure 1:**
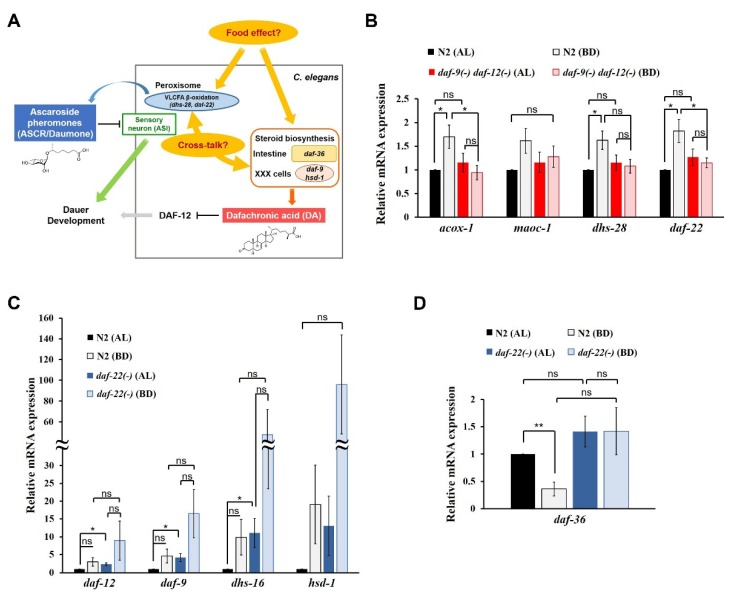
Reciprocal regulation of ASCRs and DAs by dietary cues in *C. elegans*. (**A**) A schematic diagram showing a model of reciprocal regulation between ASCRs (ascaroside pheromones) and DAs (dafachronic acid hormones) in *C. elegans*. VLCFA, very long chain fatty acid. The chemical structures of the major subtype of ASCR (ascr#1) and DA (Δ^7^-DA) are shown. (**B**) Relative mRNA expression levels of ASCR biosynthetic genes (*acox-1*, *maoc-1*, *dhs-28* and *daf-22*) in wild-type N2 and *daf-9(dh6) daf-12(rh61 rh411)* L4 worms under AL (*ad libitum* fed) and BD (bacterial deprivation) conditions. (**C**) Relative mRNA expression levels of DA biosynthetic genes (*daf-9*, *dhs-16* and *hsd-1*; *daf-12* for DA receptor) in N2 and *daf-22(ok693)* L4 worms under AL and BD conditions. (**D**) Relative mRNA expression level of DA biosynthetic gene *daf-36* in N2 and *daf-22(ok693)* L4 worms under AL and BD conditions. For (**B–D**), data are shown as mean ± SEM of four biologically independent experiments. * *p* < 0.05, ** *p* < 0.01 and ns (not significant) were determined via one-way ANOVA analysis. See the Supplementary tables for the exact *p*-values.

By contrast, under favorable conditions, early worm larvae usually undergo reproductive development during which endogenous bile acid-like steroid hormones termed dafachronic acids (DAs), cholesterol-derived ligands for DAF-12 receptor, mediate reproductive pathways [16,17,18,19,20]. Multiple DAs (e.g., Δ^7^-DA and Δ^4^-DA) are currently known, and they are synthesized via the actions of several enzymes (e.g., *daf-9*, *dhs-16*, *hsd-1* and *daf-36*) [17,18,21,22,23,24,25,26]. In particular, the serial positive feedback reactions involving DA biosynthesis lead to irreversible adult development in a DAF-12-dependent manner [20,27]. Unlike ASCRs, DA biosynthesis is usually activated under conditions of bacteria deprivation (BD), but suppressed under *ad libitum* (AL) feeding conditions [28]. The ASCR (pheromone) and DA (hormone) signaling seem to exert their functions by integrating dietary cues into developmental signals (Figure 1A) [3,21,26,27,29,30]. The dietary cues are perceived by sensory neurons that also sense ASCRs; next, these signals are coupled to neuroendocrine signaling pathways. Insulin/insulin-like growth factor-1 (IGF-1) signaling (IIS), one of the most important signaling pathways that regulates reproductive development and dauer entry, is also linked to ASCRs, DAs and the genes regulated by these small molecules [3,26,31,32,33,34]. Interestingly, ASCRs and DAs seem to have opposite effects with respect to the effects of IIS on developmental processes. For example, ASCRs inhibit IIS, whereas DAs synergizes with IIS [3,19,26,31,35].

Although the patterns of changes in the endogenous levels of ASCRs and DAs seem to be opposite during development [20,27,35] and in response to dietary cues [15,28], the molecular basis of their influences on each other remains elusive. Furthermore, it is not well understood their competitive relationship with IIS (e.g., dependence or independence), a central player in both reproductive development and dauer entry in *C. elegans*. Is not well understood. Here we report a novel reciprocal transcriptional regulatory effect between the ASCR and DA biosynthetic genes in response to dietary cues.

## 2. Results

### 2.1. Transcriptional Regulation of ASCR and DA Biosynthetic Genes by Dietary Cues

Based on the previous reports [15,27,28], we hypothesized that there may exist a crosstalk between ASCR and DA biosynthesis under certain nutritional conditions (Figure 1A). Since dietary cues seem to be highly effective in determining the choice between alternative developmental programs (i.e., dauer diapause or reproductive development [5,16]), our aim was to clarify the differential regulation of ASCR and DA biosynthetic gene transcription by specific dietary cues.

To determine whether the synthesis of either ASCR or DA influences the food intake of animal, we measured the feeding rates of wild-type N2, *daf-22(ok693)* mutant worms (defective in ASCR biosynthesis) and *daf-9(dh6) daf-12(rh61 rh411)* mutant worms (defective in both DA biosynthesis and DA signaling). To establish the different feeding conditions, we used NGM plates with OP50 OD_600_ 1.0 for the low-food condition, OD_600_ 2.5 for high-food condition, and fully saturated OP50 for the abundant condition. The feeding rate increased in proportion to the food amount; neither ASCR biosynthesis nor DA biosynthesis had any effects on the feeding rate, indicating that food intake is only influenced by food amount (Figure S1A).

Next, to optimize the nutritional conditions to show the distinct changes in ASCR or DA biosynthesis, we compared the effects of AL and BD feeding conditions. OP50 OD_600_ 2.5 was used for the AL feeding conditions; L1-synchronized worms grown under AL until they developed into L4 larvae were used under worms those conditions. For BD conditions, L1-synchronized worms were fed AL until they reached L4 stage. The L4 larvae were then shifted to unseeded NGM plates and got deprived of bacteria for 6 h. The L4 worms under AL and BD conditions were used for the assays.

First, to investigate the effects of food and DAs on ASCR biosynthesis, we examined changes in the expression levels of four ASCR biosynthetic genes (*acox-1*, *maoc-1*, *dhs-28* and *daf-22*) in L4 wild-type N2 and DA-deficient *daf-9(dh6) daf-12(rh61 rh411)* worms under AL feeding and BD conditions. ASCR gene expression was significantly induced under BD conditions compared to that under AL conditions in N2 worms, whereas the induction under BD conditions was abolished in *daf-9(dh6) daf-12(rh61 rh411)* worms (Figure 1B). This result implies that although food deprivation (BD) can stimulate the expression of ASCR biosynthetic genes, this BD-mediated stimulation of ASCR gene expression requires DA, suggesting a cross-influence between ASCRs and DAs in *C. elegans*.

Second, to evaluate the effects of diet and ASCRs on DA biosynthesis, we examined changes in the expression levels of DA biosynthetic and DA signaling genes (*daf-9*, *dhs-16* and *hsd-1*; *daf-12* for DA receptor) in L4 N2 and ASCR-deficient *daf-22(ok693)* worms under AL and BD conditions. The expression levels of the DA genes were significantly higher in *daf-22(ok693)* worms compared with those in N2 worms, indicating that the presence of ASCRs may negatively regulate the expression of DA biosynthetic genes under AL conditions (Figure 1C). Note that DA gene expression appeared to increase under BD conditions compared with that in both N2 and *daf-22(ok693)* worms, but those changes were not significant. This effect is probably due to the high variation in the results between the independent experiments. However, these results may suggest that food deprivation can induce the expression of DA biosynthetic genes. Interestingly, the expression of *daf-36*, which encodes the Rieske-like oxygenase involved in Δ^7^-desaturation of cholesterol during DA biosynthesis [22,25], was highly suppressed in N2 worms under BD conditions compared with its level under AL conditions (Figure 1D). However, in *daf-22(ok693)* worms, the suppression of *daf-36* expression was abolished under BD conditions, indicating that ASCRs are required for BD-mediated suppression of *daf-36* expression.

We also tested the effects of different amounts of food on ASCR and DA gene expression by comparing the effects of high- and low-food conditions (OP50 OD_600_ 2.5 and OD_600_ 1.0, respectively) (Figure S1B–D). The patterns of changes in the expression levels of ASCR and DA genes under high- and low-food amounts were similar to those under AL and BD conditions, although the differences were smaller (Figure S1B,C, and Figure 1B,C). In N2 worms, *daf-36* expression was significantly suppressed by decreased food abundance (Figure S1D), as previously seen (see Figure 1D). With high food abundance (OP50 OD_600_ 2.5), *daf-36* expression was significantly higher in *daf-22(ok693)* worms compared with that in N2 worms; it was not suppressed by decreased food abundance.

Taken together, these data suggest that DAs are required for the modulation of ASCR gene expression by dietary cues, by stimulating ASCR biosynthesis under mild stress conditions (e.g., BD). It is well established that ASCRs negatively regulate DA biosynthesis via IIS, but here, we have now revealed that ASCRs are required for the suppression of *daf-36* expression driven by dietary stimuli, e.g., BD conditions. For the first time, these data define a molecular basis for reciprocal regulatory effects of pheromone (ASCR) and hormone (DA) in response to dietary cues.

### 2.2. Role of IIS in the Reciprocal Regulation of Biosynthetic ASCR and DA Genes by Dietary Cues

Given that it is not well understood how IIS influences the expression of ASCR and DA genes in response to various dietary cues, we were interested in investigating how dietary cues modulate the expression of ASCR and DA biosynthetic genes and how their expression levels could be coordinated with IIS to determine the developmental fate in *C. elegans*. In addition, we were curious about the molecular mechanism underlying the influence of IIS on the reciprocal regulation of ASCR and DA biosynthetic gene expression under various dietary conditions. To this end, we measured the changes in the expression levels of ASCR and DA biosynthetic genes in the presence and absence of IIS influence.

**Figure 2:**
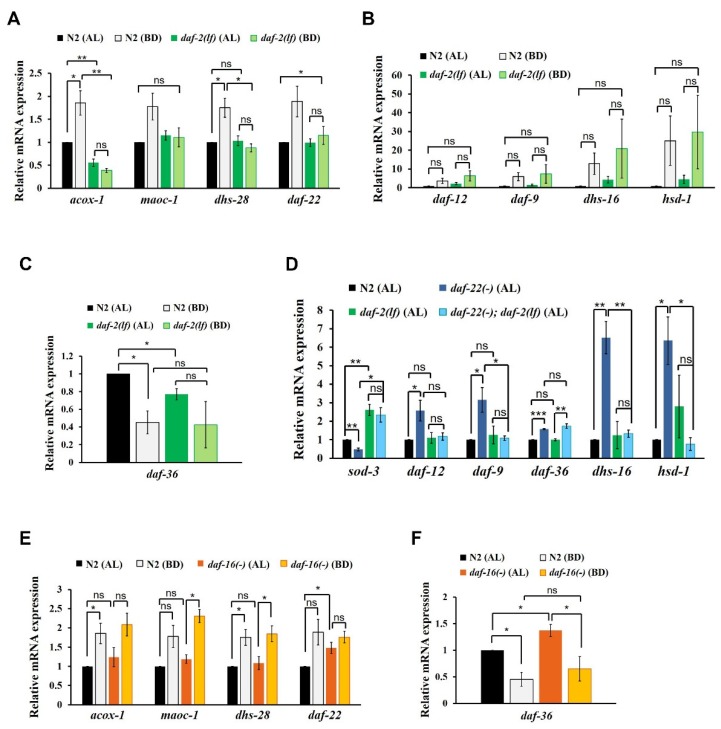
Influence of insulin/IGF-1 signaling (IIS) on the reciprocal regulation of ASCR and DA biosynthetic gene expression. (**A**) ASCR biosynthetic gene expression in N2 and *daf-2(e1370)* L4 worms under AL and BD conditions. (**B**) DA biosynthetic gene expression in N2 and *daf-2(e1370)* L4 worms under AL and BD conditions. (**C**) *daf-36* gene expression in N2 and *daf-2(e1370)* L4 worms under AL and BD conditions. (**D**) DA biosynthetic gene expression in N2, *daf-22(ok693)*, *daf-2(e1370)* and *daf-22(ok693); daf-2(e1370)* L4 worms under AL conditions at 15 °C. (**E**) ASCR biosynthetic gene expression in N2 and *daf-16(mu86)* L4 worms under AL and BD conditions. (**F**) *daf-36* gene expression in N2 and *daf-16(mu86)* L4 worms under AL and BD conditions. Data are shown as mean ± SEM of three biologically independent experiments. * *p* < 0.05, ** *p* < 0.01, ns (not significant), were determined via one-way ANOVA analysis. See the Supplementary tables for the exact *p*-values.

First, we measured the expression levels of ASCR biosynthetic genes in N2 and *daf-2(e1370)* L4 worms under AL and BD conditions. *daf-2(e1370)* worms carry a loss-of-function mutation in the *daf-2*-encoded insulin receptor, which causes a defect in IIS activity [31]. In N2 worms, the expression levels of ASCR genes significantly increased under BD conditions compared with those under AL conditions in N2 worms, whereas the BD-mediated induction of ASCR gene expression was almost abolished in *daf-2(e1370)* worms (Figure 2A). Thus, ASCR biosynthetic gene induction under BD conditions appeared to be dependent on *daf-2*/IIS function.

Second, to examine whether IIS is involved in the regulation of DA gene expression, we measured the changes in the expression levels of DA genes in N2 and *daf-2(e1370)* L4 worms. It appeared that overall DA biosynthetic gene expression increased under BD conditions in both N2 and *daf-2(e1370)* worms, but the differences were not significant (Figure 2B). As N2 and *daf-2(e1370)* worms showed similar pattern of DA gene expression changes under BD conditions, this result suggests that BD-mediated induction of DA genes is independent of *daf-2*/IIS. Under AL conditions, *daf-36* expression was significantly lower in *daf-2(e1370)* worms compared to that in N2 worms (Figure 2C), indicating that IIS may be required for *daf-36* expression in the presence of food, as previously reported [36]. The expression level of *daf-36* decreased under BD conditions relative to that under AL conditions in N2, although the change was not significant in *daf-2(e1370)* worms, suggesting that BD-mediated suppression of *daf-36* expression may partially depend on *daf-2*/IIS.

To assess the genetic epistasis between IIS and ASCR in the regulation of DA gene expression, we measured the expression levels of DA biosynthetic genes in N2, *daf-22(ok693)*, *daf-2(e1370)* and *daf-22(ok693)*; *daf-2(e1370)* L4 worms under AL food conditions. In this assay, we examined worms maintained at 15 °C, to prevent the dauer formation in the *daf-2(e1370)* animals [37]. In addition to measuring the expression levels of the DA genes, we also measured the abundance of *sod-3* mRNA as a positive control for *daf-2* function. The expression of *sod-3*, a representative target gene of the DAF-16 transcription factor (which is regulated by IIS) increased upon decreased *daf-2*/IIS activity in *daf-2(e1370)* and *daf-22(ok693)*; *daf-2(e1370)* worms. As anticipated, the expression levels of the selected DA biosynthetic genes increased in *daf-22(ok693)* worms relative to that in N2 worms (Figure 2D). However, this stimulation of DA gene expression in *daf-22(ok693)* worms was absent in *daf-22(ok693)*; *daf-2(e1370)* worms (which carry a loss-of-function *daf-2* mutation), suggesting that ASCR deficiency stimulates the expression of DA biosynthetic genes in a *daf-2*/IIS-dependent manner. This finding is also consistent with the observation that IIS activity is enhanced by ASCR deficiency and that *daf-2* is genetically downstream of *daf-22*, as *sod-3* expression is reduced in *daf-22(ok693)* worms [38]. Interestingly, only *daf-36* expression still increased in *daf-22(ok693)*; *daf-2(e1370)* worms, similar to the effect observed in *daf-22(ok693)* worms (see *daf-36* expression in Figure 2D), indicating that the increase in *daf-36* expression caused by ASCR deficiency is independent of *daf-2*/IIS. Taken together, these results suggest that the presence of ASCR negatively regulates the expression level of DA biosynthetic genes under AL conditions (see Figure 1C); this regulation seems to depend on IIS. Thus, IIS not only serves as an important signal transduction pathway, it also influences DA biosynthesis, again highlighting its crucial role in determining the developmental fate in this animal model.

Given that IIS appears to be required for the stimulation of ASCR gene expression under BD conditions (see Figure 2A), we next examined whether DAF-16, a FOXO transcription factor regulated by IIS, is required for the modulation of ASCR or DA gene expression in response to dietary cues [32]. We first measured the mRNA expression levels of ASCR biosynthetic genes in N2 and *daf-16(mu86)* L4 worms under AL and BD conditions. We found that the expression level of ASCR genes increased under BD conditions compared to those under AL conditions in both N2 and *daf-16(mu86)* worms (Figure 2E). There were no significant differences in the levels of ASCR gene expression levels between N2 and *daf-16(mu86)* worms under AL conditions, except for *daf-22*. This result indicates that ASCR induction under BD conditions may be independent of *daf-16*, despite being IIS-dependent, suggesting that other factors downstream of *daf-2*/IIS may be involved in ASCR gene regulation by dietary cues.

Next, we examined whether DAF-16 regulates DA gene expression, by measuring the expression levels of the DA genes in N2 and *daf-16(mu86)* worms under AL and BD conditions. The expression level of DA genes increased under BD conditions compared to those under AL conditions in both N2 and *daf-16(mu86)* worms; however none of these changes were not significant (Figure S1E). There seemed to be no significant differences in the expression levels of DA genes in N2 and *daf-16(mu86)* worms under both AL and BD conditions, suggesting that DAF-16 does not regulate the expression levels of at least some genes related to DA signaling and biosynthesis (*daf-12*, *daf-9*, *dhs-16* and *hsd-1*). Furthermore, the expression level of *daf-36* was significantly lower under BD conditions compared with that under AL conditions in both N2 and *daf-16(mu86)* worms, although it was slightly higher in *daf-16(mu86)* worms compared to that in N2 worms under AL conditions (Figure 2F). These results indicate that food deprivation suppresses *daf-36* expression, independently of IIS and *daf-16*. Apparently, *daf-16* may partially inhibit *daf-36* expression in the presence of abundant food, which is consistent with a previous report [36].

## 3. Discussion

In this study, we present a novel molecular basis for the reciprocal regulation between ASCR and DA biosynthetic genes. Our study of this competitive regulatory interplay between pheromone (ASCR) and hormone (DA) in the presence of different dietary cues that can influence development would enhance our understanding of the impact of nutritional stress on *C. elegans* life history. Especially during the development, the reciprocal regulatory effects of ASCRs and DAs in coordination with IIS, in which dietary cues may function, appear to determine the choice of either continuous reproductive development or entry into dauer diapause.

**Figure 3:**
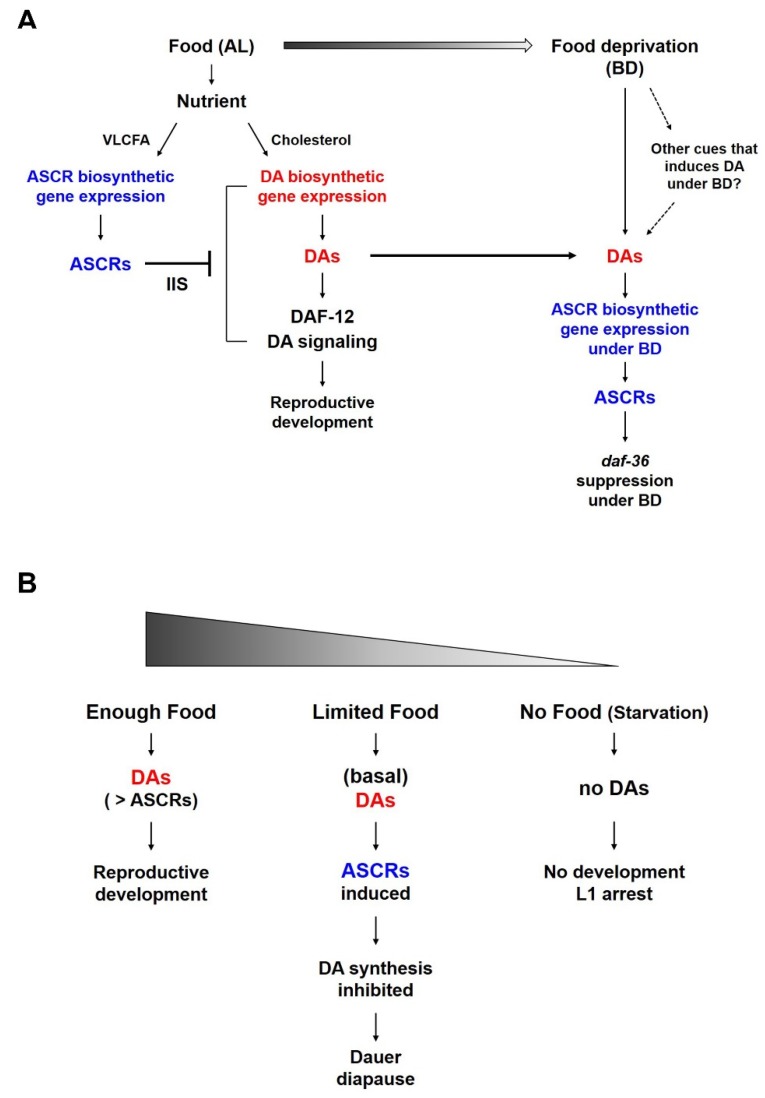
A working model of the reciprocal regulation between ASCR and DA and their effects on *C. elegans* life history under different nutritional conditions. (**A**) Reciprocal regulation between ASCR and DA in L4 worms in response to food (AL) and under food deprivation conditions (BD). Different nutritional conditions influence ASCR and DA synthesis, eventually coordinating environmental cues (food availability) and animal development or survival. (**B**) Development of L1/L2 worms in response to different nutritional conditions. In both (**A**,**B**), the color gradient represents the amount of food.

It is well known that ASCRs negatively regulate DA synthesis and DA signaling (Figure 3A). Under food-abundant conditions, modified by the AL feeding conditions in this study, DAs and DA signaling promote dauer bypass in L2 worms, which then proceed through reproductive development. ASCRs can inhibit DA synthesis and DA signaling via the action of IIS, eventually inhibiting the reproductive development and inducing dauer formation. However, as shown in Figure 3A, when the worms grown under AL conditions were shifted to BD conditions (thus experiencing food deprivation), the DAs previously synthesized under AL conditions might still exist and these DAs could then stimulate the expression of ASCR gene expression. This higher ASCR level could suppress *daf-36* expression under food deprivation conditions, in a *daf-2*/IIS-independent manner. We do not exclude the possibility that there may exist other cues that could induce or maintain gene expression under BD conditions may exist, and this possibility requires further studies.

The result that ASCR biosynthesis increases under BD conditions appears to be contradictory to a previous report [15] in which ASCR concentration in the worm body was shown to increase with more food. However, this discrepancy can be explained by difference in the experimental methods used in the two studies. Joo et al., used worms that were constantly fed a specific amount of food as they developed from L1 larvae into adult, there was no change of food amount within the same group of worms. In this study, both the AL and BD groups started to grow with the same amount of food until the worms in the BD group were briefly deprived of food (i.e., for six hours) while the food level of the AL groups remained constant. This method not only allows worms to develop well without dauer entry or starvation-induced developmental arrest before the experiment, but also shows the distinct changes between the AL and BD groups. Thus, the BD conditions can be interpreted as a mild stress relative to the effects of starvation or *ad libitum* feeding. Moreover, this phenomenon is consistent in part with a previous study that decreased DA production in the XXX cells (which is known to perform a neuroendocrine secretary function in larvae) activates a positive feedback loop through *daf-9* in the hypodermis. This causes increase in DA levels that is sufficient to allow the worms to bypass dauer entry and to continue into reproductive development even under mild stress conditions [20,21,26,27].

Both ASCRs and DAs are important molecules in the regulation of development, survival and aging in young worm larvae, especially in relation to dauer development. Food availability is also crucial for controlling developmental decision; there are several larval developmental processes are influenced by food availability (Figure 3B). For instance, (i) when there is enough food and the concentration of DAs is higher than that of ASCRs, worms can proceed reproductive development. (ii) When there is a limited amount of food (i.e., enough food to support L1 growth to molting into L2 larvae, but not enough to support full reproductive development), there would be only a basal level of DAs is produced due to the food limitation. This basal level of DAs can induce the ASCRs biosynthesis under BD conditions, which may result in the inhibition of DA biosynthesis (i.e., *daf-36* suppression under BD conditions), as well as the entry into dauer diapause. (iii) When eggs hatch in an environment completely devoid of food, L1 worms do not have sufficient nutrients enough to develop or molt into L2 larvae and they instead arrest in L1 stage, *i.e*., L1 arrest.

It is interesting to note that positive cross-regulatory interactions between hormone and pheromone activity that differs from the negative competitive relationship between ASCRs and DAs in *C. elegans* have been previously reported. During ovulation in female mice, sex hormone-regulated egg release was found to release pheromones to attract males [39]. Furthermore, the ability of female mice to detect attractive male pheromones depends on their ovulation status, which is signaled by a sex hormone [40], highlighting a functional synergistic crosstalk between hormones and pheromones. These examples raise the possibility that *C. elegans* may have similar positive cross-regulation between these two small molecules as a part of some metabolic pathways or in other functions, such as germ cell development, neuronal network formation and aging.

## 4. Materials and Methods

### 4.1. Maintenance of C. elegans Strains

The *C. elegans* strains used in this work were N2 Bristol (wild-type), RB859 *daf-22(ok693)* II, AA161 *daf-9(dh6) daf-12(rh61 rh411)* X, CB1370 *daf-2(e1370)* III, CF1038 *daf-16(mu86)* I, YP0025 *daf-22(ok693)* II; *daf-2(e1370)* III mutant worms. All animals were cultured as previously described [41]. Worms were maintained at 20 °C on nematode growth medium (NGM) agar plates, and fed *Escherichia coli* strain OP50 grown in 2xYT medium. For the experiments using the YP0025 strain, worms were grown at 15 °C.

### 4.2. Worm Sample Preparation

Age-synchronized worms were cultured on NGM agar plates containing equal amounts of OP50. To prepare specific quantities of OP50, a single colony of *E. coli* OP50 was cultured in 2xYT medium for overnight (~16 h) at 37 °C, harvested and washed three times in S-basal medium. The amount of OP50 was determined by measuring the optical density (OD) at 600 nm with a UV spectrophotometer (Beckman, Brea, CA, USA). To make AL plates, 2 mL of OP50 suspension (at a concentration of OD_600_ 2.5) was spread on 90-mm NGM plates without peptone and the plates were air-dried in a clean bench and stored at 4 °C until use. To avoid crowding and starvation, two thousand L1-synchronized worms were obtained and grown on one AL plate until they reached young adult stage. They were then harvested using distilled water into 1.5-mL conical tubes. For the BD conditions, worms were grown on AL (OD_600_ 2.5) plates identically to the worms grown under AL conditions until they reached the L4 stage. They were then washed with S-basal medium to remove the bacteria and transferred to unseeded (i.e., no *E. coli*) NGM agar plates with no peptone. After 6 h of bacterial deprivation on the unseeded plates, young adult worms were harvested using distilled water. For experiments using the high and low food conditions, OD_600_ 2.5 and OD_600_ 1.0 plates were used, respectively.

### 4.3. Quantitative Real-Time Polymerase Chain Reaction (qRT-PCR)

Total RNA was extracted from worm samples using TRIzol Reagent (Invitrogen, Carlsbad, CA, USA) (Thermo Fisher Scientific, Waltham, MA, USA) and purified using the RNeasy kit (QIAGEN, Hilden, Germany). The RNA was reverse-transcribed using a Transcriptor First Strand cDNA Synthesis Kit (Roche, Basel, Switzerland) with oligo-dT priming. qRT-PCR was performed using iQ SYBR Green Supermix (Bio-Rad Laboratories, Hercules, CA, USA) according to the manufacturer’s instructions. The reaction products were analyzed using a CFX Connect^TM^ Real-Time PCR Detection System (Bio-Rad Laboratories, Hercules, CA, USA). Relative mRNA expression was determined using the ΔΔ*C*_T_ method; the mRNA expression level of *act-1* was used as a reference to normalize the results. The sequences of the primers used for qRT-PCR are listed in Table S1.

### 4.4. Feeding Rate Assay (Food Intake Assay)

Feeding rate was measured by counting pharyngeal pumping, as previously described [42]. For each animal (*n* = 10 for each genotype) and each food condition (OD_600_ 1.0, OD_600_ 2.5, abundant food [i.e., OP50 fully grown and saturated in 2xYT medium]), the rhythmic contractions of the pharyngeal bulb were counted over a 10-s period under a microscope.

### 4.5. Statistics

The error bars of all results represent the standard error of the mean (SEM). For the results of qRT-PCR analyses, one-way ANOVA with Tukey’s post-test was used for the statistics. Detailed values for the results of the experiments including the exact *p*-values are presented in the Supplementary Materials (Supplementary Tables S2–S10). For the feeding rate assay, we used Two-Way ANOVA analysis with Bonferroni post-test for the statistics.

## 5. Conclusions

In this study, our work defines a molecular basis for a novel reciprocal gene regulation of animal pheromones and hormones to cope with stressful conditions during development and aging. The reciprocal regulation between ASCR and DA biosynthetic genes appears to be linked directly or indirectly to IIS. In the crosstalk between these two small molecules, it appears that the presence of ASCR determines the IIS-dependency of DA gene expression regardless of dietary conditions. Collectively, this work provides an important insight into how *C. elegans* regulates developmental processes under different environmental stresses. The results may help clarify how the complex molecular interplay between these highly studied cellular signaling pathways, both of which are known to control numerous important responses to environmental stress, can promote survival in animals—perhaps even in humans.

## Supplementary Materials

Supplementary materials can be found at https://www.mdpi.com/1422-0067/21/7/2366/s1.

## Abbreviations

AL	*ad libitum*
ASCR	ascaroside
BD	bacterial deprivation
DA	dafachronic acid
IGF-1 IIS OD	insulin-like growth factor-1 insulin/IGF-1 signaling optical density
VLCFA	very long chain fatty acid
