# Supplementary material for: A Molecular Basis for Reciprocal Regulation between Pheromones and Hormones in Response to Dietary Cues in C. elegans
Source: Int J Mol Sci. 2020 Mar 29;21(7):2366. doi: 10.3390/ijms21072366 (PMC7177881; doi:10.3390/ijms21072366)
Supplement: Supplementary file 1 [file ijms-21-02366-s001.pdf]

Supplementary Materials

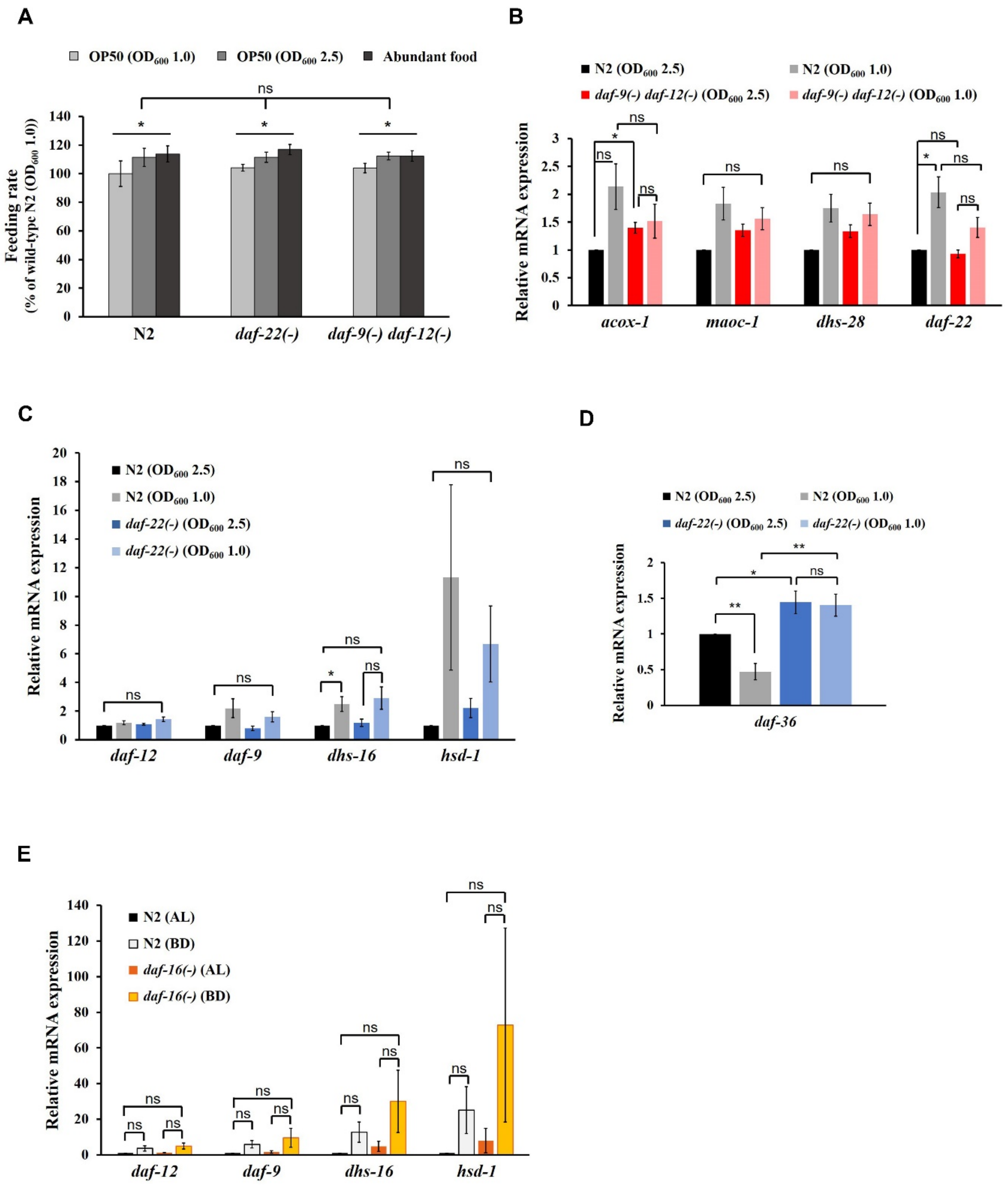

Figure S1. Reciprocal regulation of ASCR and DA gene expression with different amounts of food. (A) Food intake rates of N2, *daf-22(ok693)* and *daf-9(dh6) daf-12(rh61 rh411)* worms with different food levels OP50 (OD_{600} 1.0), OP50 (OD_{600} 2.5) and abundant food (OP50 fully

grown and saturated in 2xYT). Data are expressed as a percentage of N2 (OD_{600} 1.0). Data are shown as mean ± SEM of four independent experiments (n=10). * $P = 0.0175$, two-way ANOVA analysis. **(B)** ASCR biosynthetic gene expression in N2 and *daf-9(dh6) daf-12(rh61 rh411)* worms that had been fed with high OP50 (OD_{600} 2.5) or low OP50 (OD_{600} 1.0) food levels. Data are shown as mean ± SEM of three biologically independent experiments. * $P < 0.05$ and ns (not significant) were determined by one-way ANOVA analysis. The exact *P*-values are reported in Supplementary Table S4. **(C)** DA biosynthetic gene expression in N2 and *daf-22(ok693)* worms that had been fed with high OP50 (OD_{600} 1.0) or low OP50 (OD_{600} 1.0) food levels. **(D)** *daf-36* gene expression in N2 and *daf-22(ok693)* fed with high OP50 (OD_{600} 1.0) or low OP50 (OD_{600} 1.0) food levels. For **(C)** and **(D)**, data are shown as mean ± SEM of four biologically independent experiments. * $P < 0.05$, ** $P < 0.01$ and ns (not significant) were determined by one-way ANOVA analysis. The exact *P*-values are reported in Supplementary Table S5. **(E)** DA biosynthetic gene expression in N2 and *daf-16(mu86)* L4 worms under AL and BD conditions. Data are shown as mean ± SEM of three biologically independent experiments. * $P < 0.05$, ** $P < 0.01$ and ns (not significant) were determined by one-way ANOVA analysis. The exact *P*-values are reported in Supplementary Table S10.

Table S1. Nucleotide sequence of primers used in qRT-PCR

Target Gene	Primer Sequence
act-1	(Forward) GTATGGAGTCCGCCGGA<br>(Reverse) CTTCATGGTTGATGGGGCAA
acox-1	(Forward) ACTCATGCGATGGCTGCACAG<br>(Reverse) CGCTCGAATGAGCCACTTGGCT
maoc-1	(Forward) TGGGATCAGGAGATATGAATCC<br>(Reverse) CCTCGTGCAAGTCAATAAAGG
dhs-28	(Forward) CTAGACTTACCGAGACTGTCATGC<br>(Reverse) CAGAAGAAGCACCAGAAGAAGC
daf-22	(Forward) AAGTGGAAACTCCGATGTGG<br>(Reverse) TCTTAGGGGCATTGATCACC
daf-12	(Forward) GTTCGCATCGTTACAGAACGA<br>(Reverse) TTCTCCTGGCAGCTCTTCG
daf-9	(Forward) GAACTGTTACCCATGGCTTCGT<br>(Reverse) CACCTTGATGCCGGTTGACG
daf-36	(Forward) GAGCAGCATGGTCCTGGAAT<br>(Reverse) CATTGCTTCCACTGTCATGAAG
dhs-16	(Forward) TGCCGTTGAAGCCTATATGGAT<br>(Reverse) GCTTCCCAAGCACGCTCG
hsd-1	(Forward) AATCGTGTTAGTGGATGTGCTG<br>(Reverse) CCATGCAAGCTTTACGGTCAC
sod-3	(Forward) AGCTGATGGACACTATTAAGCG<br>(Reverse) CACAGGTGGCGATCTTCAAG

Table S2. The relative expression levels of ASCR biosynthetic genes in N2 and *daf-9(dh6) daf-12(rh61 rh411)* under AL and BD conditions
(Related to Figure 1B)

Gene	Relative mRNA expression (fold ± SEM)				*P*-value (analyzed by One-way ANOVA)			
	N2 (AL)	N2 (BD)	*daf-9(-) daf-12(-)* (AL)	*daf-9(-) daf-12(-)* (BD)	Group	Comparison		
acox-1	1.00±0.00	1.70±0.25	1.15±0.20	0.94±0.16	0.039	N2 (AL)	N2 (BD)	0.030
						N2 (AL)	*daf-9(-) daf-12(-)* (AL)	0.464
						daf-9(-) daf-12(-) (AL)	*daf-9(-) daf-12(-)* (BD)	0.432
						N2 (BD)	*daf-9(-) daf-12(-)* (BD)	0.041
maoc-1	1.00±0.00	1.62±0.26	1.16±0.21	1.28±0.23	0.229	N2 (AL)	N2 (BD)	0.057
						N2 (AL)	*daf-9(-) daf-12(-)* (AL)	0.485
						daf-9(-) daf-12(-) (AL)	*daf-9(-) daf-12(-)* (BD)	0.713
						N2 (BD)	*daf-9(-) daf-12(-)* (BD)	0.367
dhs-28	1.00±0.00	1.63±0.19	1.15±0.16	1.08±0.15	0.042	N2 (AL)	N2 (BD)	0.018
						N2 (AL)	*daf-9(-) daf-12(-)* (AL)	0.386
						daf-9(-) daf-12(-) (AL)	*daf-9(-) daf-12(-)* (BD)	0.744
						N2 (BD)	*daf-9(-) daf-12(-)* (BD)	0.064
daf-22	1.00±0.00	1.82±0.24	1.27±0.18	1.15±0.10	0.017	N2 (AL)	N2 (BD)	0.015
						N2 (AL)	*daf-9(-) daf-12(-)* (AL)	0.176
						daf-9(-) daf-12(-) (AL)	*daf-9(-) daf-12(-)* (BD)	0.571
						N2 (BD)	*daf-9(-) daf-12(-)* (BD)	0.044

Table S3. The relative expression of DA biosynthetic genes in N2 and *daf-22(ok693)* under AL and BD conditions
(Related to Figure 1C, 1D)

Gene	Relative mRNA expression (fold ± SEM)				*P*-value (analyzed by One-way ANOVA)			
	N2 (AL)	N2 (BD)	*daf-22(-)* (AL)	*daf-22(-)* (BD)	Group	Comparison		
daf-12	1.00±0.00	2.96±1.20	2.28±0.36	8.93±5.44	0.243	N2 (AL)	N2 (BD)	0.153
						N2 (AL)	*daf-22(-)* (AL)	0.012
						daf-22(-) (AL)	*daf-22(-)* (BD)	0.268
						N2 (BD)	*daf-22(-)* (BD)	0.325
daf-9	1.00±0.00	4.62±1.96	4.16±1.12	16.51±6.81	0.045	N2 (AL)	N2 (BD)	0.114
						N2 (AL)	*daf-22(-)* (AL)	0.030
						daf-22(-) (AL)	*daf-22(-)* (BD)	0.123
						N2 (BD)	*daf-22(-)* (BD)	0.144
dhs-16	1.00±0.00	9.86±5.00	11.06±4.08	47.61±24.14	0.090	N2 (AL)	N2 (BD)	0.127
						N2 (AL)	*daf-22(-)* (AL)	0.049
						daf-22(-) (AL)	*daf-22(-)* (BD)	0.186
						N2 (BD)	*daf-22(-)* (BD)	0.177
hsd-1	1.00±0.00	19.08±11.05	13.09±8.35	96.06±47.66	0.071	N2 (AL)	N2 (BD)	0.153
						N2 (AL)	*daf-22(-)* (AL)	0.198
						daf-22(-) (AL)	*daf-22(-)* (BD)	0.137
						N2 (BD)	*daf-22(-)* (BD)	0.167
daf-36	1.00±0.00	0.36±0.13	1.41±0.28	1.42±0.43	0.049	N2 (AL)	N2 (BD)	0.002
						N2 (AL)	*daf-22(-)* (AL)	0.190
						daf-22(-) (AL)	*daf-22(-)* (BD)	0.993
						N2 (BD)	*daf-22(-)* (BD)	0.057

Table S4. The relative expression levels of ASCR biosynthetic genes in N2 and *daf-9(dh6) daf-12(rh61 rh411)* under OD_{600} 2.5 and OD_{600} 1.0 growth conditions
(Related to Figure S1B)

Gene	Relative mRNA expression (fold ± SEM)				*P*-value (analyzed by One-way ANOVA)			
	N2 (OD 2.5)	N2 (OD 1.0)	*daf-9(-) daf-12(-)* (OD 2.5)	*daf-9(-) daf-12(-)* (OD 1.0)	Group	Comparison		
acox-1	1.00±0.00	2.14±0.47	1.40±0.11	1.52±0.35	0.136	N2 (OD 2.5)	N2 (OD 1.0)	0.073
						N2 (OD 2.5)	*daf-9(-) daf-12(-)* (OD 2.5)	0.024
						daf-9(-) daf-12(-) (OD 2.5)	*daf-9(-) daf-12(-)* (OD 1.0)	0.762
						N2 (OD 1.0)	*daf-9(-) daf-12(-)* (OD 1.0)	0.352
maoc-1	1.00±0.00	1.83±0.34	1.35±0.13	1.56±0.23	0.115	N2 (OD 2.5)	N2 (OD 1.0)	0.070
						N2 (OD 2.5)	*daf-9(-) daf-12(-)* (OD 2.5)	0.052
						daf-9(-) daf-12(-) (OD 2.5)	*daf-9(-) daf-12(-)* (OD 1.0)	0.465
						N2 (OD 1.0)	*daf-9(-) daf-12(-)* (OD 1.0)	0.542
dhs-28	1.00±0.00	1.75±0.29	1.34±0.13	1.64±0.24	0.101	N2 (OD 2.5)	N2 (OD 1.0)	0.060
						N2 (OD 2.5)	*daf-9(-) daf-12(-)* (OD 2.5)	0.061
						daf-9(-) daf-12(-) (OD 2.5)	*daf-9(-) daf-12(-)* (OD 1.0)	0.322
						N2 (OD 1.0)	*daf-9(-) daf-12(-)* (OD 1.0)	0.783
daf-22	1.00±0.00	2.04±0.32	0.93±0.08	1.40±0.21	0.013	N2 (OD 2.5)	N2 (OD 1.0)	0.031
						N2 (OD 2.5)	*daf-9(-) daf-12(-)* (OD 2.5)	0.417
						daf-9(-) daf-12(-) (OD 2.5)	*daf-9(-) daf-12(-)* (OD 1.0)	0.100
						N2 (OD 1.0)	*daf-9(-) daf-12(-)* (OD 1.0)	0.170

Table S5. The relative expression levels of DA biosynthetic genes in N2 and *daf-22(ok693)* under OD_{600} 2.5 and OD_{600} 1.0 growth conditions
(Related to Figure S1C, S1D)

Gene	Relative mRNA expression (fold ± SEM)				*P*-value (analyzed by One-way ANOVA)			
	N2 (OD 2.5)	N2 (OD 1.0)	*daf-22(-)* (OD 2.5)	*daf-22(-)* (OD 1.0)	Group	Comparison		
daf-12	1.00±0.00	1.19±0.15	1.09±0.07	1.43±0.18	0.121	N2 (OD 2.5)	N2 (OD 1.0)	0.253
						N2 (OD 2.5)	*daf-22(-)* (OD 2.5)	0.258
						daf-22(-) (OD 2.5)	*daf-22(-)* (OD 1.0)	0.126
						N2 (OD 1.0)	*daf-22(-)* (OD 1.0)	0.324
daf-9	1.00±0.00	2.19±0.74	0.80±0.16	1.60±0.40	0.142	N2 (OD 2.5)	N2 (OD 1.0)	0.156
						N2 (OD 2.5)	*daf-22(-)* (OD 2.5)	0.260
						daf-22(-) (OD 2.5)	*daf-22(-)* (OD 1.0)	0.113
						N2 (OD 1.0)	*daf-22(-)* (OD 1.0)	0.503
dhs-16	1.00±0.00	2.49±0.57	1.19±0.30	2.90±0.88	0.072	N2 (OD 2.5)	N2 (OD 1.0)	0.040
						N2 (OD 2.5)	*daf-22(-)* (OD 2.5)	0.557
						daf-22(-) (OD 2.5)	*daf-22(-)* (OD 1.0)	0.113
						N2 (OD 1.0)	*daf-22(-)* (OD 1.0)	0.707
hsd-1	1.00±0.00	11.33±7.23	2.21±0.75	6.68±2.95	0.281	N2 (OD 2.5)	N2 (OD 1.0)	0.203
						N2 (OD 2.5)	*daf-22(-)* (OD 2.5)	0.158
						daf-22(-) (OD 2.5)	*daf-22(-)* (OD 1.0)	0.192
						N2 (OD 1.0)	*daf-22(-)* (OD 1.0)	0.574
daf-36	1.00±0.00	0.47±0.13	1.45±0.18	1.41±0.17	0.001	N2 (OD 2.5)	N2 (OD 1.0)	0.006
						N2 (OD 2.5)	*daf-22(-)* (OD 2.5)	0.046
						daf-22(-) (OD 2.5)	*daf-22(-)* (OD 1.0)	0.877
						N2 (OD 1.0)	*daf-22(-)* (OD 1.0)	0.005

Table S6. The relative expression levels of ASCR biosynthetic genes in N2 and *daf-2(e1370)* under AL and BD conditions
(Related to Figure 2A)

Gene	Relative mRNA expression (fold ± SEM)				*P*-value (analyzed by One-way ANOVA)			
	N2 (AL)	N2 (BD)	*daf-2(-)* (AL)	*daf-2(-)* (BD)	Group	Comparison		
acox-1	1.00±0.00	1.86±0.26	0.56±0.08	0.39±0.05	0.0003	N2 (AL)	N2 (BD)	0.031
						N2 (AL)	*daf-2(-)* (AL)	0.005
						daf-2(-) (AL)	*daf-2(-)* (BD)	0.137
						N2 (BD)	*daf-2(-)* (BD)	0.005
maoc-1	1.00±0.00	1.78±0.29	1.15±0.10	1.11±0.20	0.064	N2 (AL)	N2 (BD)	0.055
						N2 (AL)	*daf-2(-)* (AL)	0.210
						daf-2(-) (AL)	*daf-2(-)* (BD)	0.857
						N2 (BD)	*daf-2(-)* (BD)	0.132
dhs-28	1.00±0.00	1.76±0.21	1.04±0.11	0.88±0.09	0.004	N2 (AL)	N2 (BD)	0.023
						N2 (AL)	*daf-2(-)* (AL)	0.756
						daf-2(-) (AL)	*daf-2(-)* (BD)	0.320
						N2 (BD)	*daf-2(-)* (BD)	0.018
daf-22	1.00±0.00	1.89±0.33	0.99±0.64	1.15±0.32	0.035	N2 (AL)	N2 (BD)	0.055
						N2 (AL)	*daf-2(-)* (AL)	0.929
						daf-2(-) (AL)	*daf-2(-)* (BD)	0.493
						N2 (BD)	*daf-2(-)* (BD)	0.127

Table S7. The relative expression levels of DA biosynthetic genes in N2 and *daf-2(e1370)* under AL and BD conditions
(Related to Figure 2B, 2C)

Gene	Relative mRNA expression (fold ± SEM)				*P*-value (analyzed by One-way ANOVA)			
	N2 (AL)	N2 (BD)	*daf-2(-)* (AL)	*daf-2(-)* (BD)	Group	Comparison		
daf-12	1.00±0.00	3.63±1.41	2.20±0.62	6.34±2.72	0.170	N2 (AL)	N2 (BD)	0.137
						N2 (AL)	*daf-2(-)* (AL)	0.123
						daf-2(-) (AL)	*daf-2(-)* (BD)	0.212
						N2 (BD)	*daf-2(-)* (BD)	0.426
daf-9	1.00±0.00	5.91±2.08	1.59±0.50	7.34±4.93	0.316	N2 (AL)	N2 (BD)	0.078
						N2 (AL)	*daf-2(-)* (AL)	0.377
						daf-2(-) (AL)	*daf-2(-)* (BD)	0.304
						N2 (BD)	*daf-2(-)* (BD)	0.803
dhs-16	1.00±0.00	12.81±5.71	4.32±1.83	20.88±15.67	0.392	N2 (AL)	N2 (BD)	0.107
						N2 (AL)	*daf-2(-)* (AL)	0.143
						daf-2(-) (AL)	*daf-2(-)* (BD)	0.353
						N2 (BD)	*daf-2(-)* (BD)	0.654
hsd-1	1.00±0.00	25.05±13.15	4.50±2.20	29.74±19.61	0.292	N2 (AL)	N2 (BD)	0.142
						N2 (AL)	*daf-2(-)* (AL)	0.188
						daf-2(-) (AL)	*daf-2(-)* (BD)	0.270
						N2 (BD)	*daf-2(-)* (BD)	0.852
daf-36	1.00±0.00	0.45±0.13	0.77±0.06	0.43±0.26	0.076	N2 (AL)	N2 (BD)	0.013
						N2 (AL)	*daf-2(-)* (AL)	0.022
						daf-2(-) (AL)	*daf-2(-)* (BD)	0.272
						N2 (BD)	*daf-2(-)* (BD)	0.933

Table S8. The relative expression levels of DA biosynthetic genes in N2, *daf-22(ok693), daf-2(e1370),* and *daf-22(ok693);daf-2(e1370)* under AL conditions
(Related to Figure 2D)

Gene	Relative mRNA expression (fold ± SEM)				*P*-value (analyzed by One-way ANOVA)			
	N2 (AL)	daf-22(-) (AL)	*daf-2(-)* (AL)	*daf-22(-); daf-2(-)* (AL)	Group	Comparison		
sod-3	1.00±0.00	3.63±1.41	2.20±0.62	6.34±2.72	0.0008	N2	*daf-22(-)*	0.003
						N2	*daf-2(-)*	0.006
						daf-2(-)	*daf-22(-); daf-2(-)*	0.615
						daf-22(-)	*daf-22(-); daf-2(-)*	0.010
daf-12	1.00±0.00	3.63±1.41	2.20±0.62	6.34±2.72	0.029	N2	*daf-22(-)*	0.047
						N2	*daf-2(-)*	0.742
						daf-2(-)	*daf-22(-); daf-2(-)*	0.844
						daf-22(-)	*daf-22(-); daf-2(-)*	0.076
daf-9	1.00±0.00	5.91±2.08	1.59±0.50	7.34±4.93	0.017	N2	*daf-22(-)*	0.031
						N2	*daf-2(-)*	0.616
						daf-2(-)	*daf-22(-); daf-2(-)*	0.740
						daf-22(-)	*daf-22(-); daf-2(-)*	0.037
daf-36	1.00±0.00	12.81±5.71	4.32±1.83	20.88±15.67	0.00005	N2	*daf-22(-)*	0.00005
						N2	*daf-2(-)*	0.999
						daf-2(-)	*daf-22(-); daf-2(-)*	0.005
						daf-22(-)	*daf-22(-); daf-2(-)*	0.261
dhs-16	1.00±0.00	25.05±13.15	4.50±2.20	29.74±19.61	0.0004	N2	*daf-22(-)*	0.003
						N2	*daf-2(-)*	0.753
						daf-2(-)	*daf-22(-); daf-2(-)*	0.921
						daf-22(-)	*daf-22(-); daf-2(-)*	0.004
hsd-1	1.00±0.00	0.45±0.13	0.77±0.06	0.43±0.26	0.044	N2	*daf-22(-)*	0.023
						N2	*daf-2(-)*	0.411
						daf-2(-)	*daf-22(-); daf-2(-)*	0.368
						daf-22(-)	*daf-22(-); daf-2(-)*	0.022

Table S9. The relative expression levels of ASCR biosynthetic genes in N2 and *daf-16(mu86)* under AL and BD conditions
(Related to Figure 2E)

Gene	Relative mRNA expression (fold ± SEM)				*P*-value (analyzed by One-way ANOVA)			
	N2 (AL)	N2 (BD)	*daf-16(-)* (AL)	*daf-16(-)* (BD)	Group	Comparison		
acox-1	1.00±0.00	1.86±0.26	1.24±0.25	2.09±0.30	0.034	N2 (AL)	N2 (BD)	0.031
						N2 (AL)	*daf-16(-)* (AL)	0.388
						daf-16(-) (AL)	*daf-16(-)* (BD)	0.092
						N2 (BD)	*daf-16(-)* (BD)	0.594
maoc-1	1.00±0.00	1.78±0.29	1.19±0.11	2.31±1.57	0.003	N2 (AL)	N2 (BD)	0.055
						N2 (AL)	*daf-16(-)* (AL)	0.166
						daf-16(-) (AL)	*daf-16(-)* (BD)	0.005
						N2 (BD)	*daf-16(-)* (BD)	0.193
dhs-28	1.00±0.00	1.76±0.21	1.09±0.17	1.85±0.21	0.014	N2 (AL)	N2 (BD)	0.023
						N2 (AL)	*daf-16(-)* (AL)	0.633
						daf-16(-) (AL)	*daf-16(-)* (BD)	0.045
						N2 (BD)	*daf-16(-)* (BD)	0.761
daf-22	1.00±0.00	1.89±0.33	1.48±0.15	1.76±0.15	0.051	N2 (AL)	N2 (BD)	0.055
						N2 (AL)	*daf-16(-)* (AL)	0.031
						daf-16(-) (AL)	*daf-16(-)* (BD)	0.252
						N2 (BD)	*daf-16(-)* (BD)	0.746

Table S10. The relative expression levels of DA biosynthetic genes in N2 and *daf-16(mu86)* under AL and BD conditions
(Related to Figure S1E, 2F)

Gene	Relative mRNA expression (fold ± SEM)				*P*-value (analyzed by One-way ANOVA)			
	N2 (AL)	N2 (BD)	*daf-16(-)* (AL)	*daf-16(-)* (BD)	Group	Comparison		
daf-12	1.00±0.00	3.63±1.41	1.18±0.13	4.91±1.64	0.09	N2 (AL)	N2 (BD)	0.137
						N2 (AL)	*daf-16(-)* (AL)	0.249
						daf-16(-) (AL)	*daf-16(-)* (BD)	0.086
						N2 (BD)	*daf-16(-)* (BD)	0.585
daf-9	1.00±0.00	5.91±2.08	1.61±0.76	9.57±5.29	0.200	N2 (AL)	N2 (BD)	0.078
						N2 (AL)	*daf-16(-)* (AL)	0.465
						daf-16(-) (AL)	*daf-16(-)* (BD)	0.211
						N2 (BD)	*daf-16(-)* (BD)	0.555
dhs-16	1.00±0.00	12.81±5.71	4.79±2.78	30.04±17.46	0.204	N2 (AL)	N2 (BD)	0.107
						N2 (AL)	*daf-16(-)* (AL)	0.245
						daf-16(-) (AL)	*daf-16(-)* (BD)	0.226
						N2 (BD)	*daf-16(-)* (BD)	0.401
hsd-1	1.00±0.00	25.05±13.15	8.00±6.84	72.92±54.40	0.334	N2 (AL)	N2 (BD)	0.142
						N2 (AL)	*daf-16(-)* (AL)	0.364
						daf-16(-) (AL)	*daf-16(-)* (BD)	0.302
						N2 (BD)	*daf-16(-)* (BD)	0.441
daf-36	1.00±0.00	0.45±0.13	1.37±0.11	0.65±0.23	0.008	N2 (AL)	N2 (BD)	0.013
						N2 (AL)	*daf-16(-)* (AL)	0.031
						daf-16(-) (AL)	*daf-16(-)* (BD)	0.048
						N2 (BD)	*daf-16(-)* (BD)	0.490
